# Coming of age in the frontal cortex: The role of puberty in cortical maturation

**DOI:** 10.1016/j.semcdb.2021.04.021

**Published:** 2021-05-10

**Authors:** Kristen Delevich, Madeline Klinger, Nana J. Okada, Linda Wilbrecht

**Affiliations:** a Department of Psychology, University of California, Berkeley, CA 94720, USA; b Helen Wills Neuroscience Institute, University of California, Berkeley, CA 94720, USA

**Keywords:** Puberty, mPFC, Estradiol, Testosterone, Synaptic pruning, PV interneurons, Dopamine

## Abstract

Across species, adolescence is a period of growing independence that is associated with the maturation of cognitive, social, and affective processing. Reorganization of neural circuits within the frontal cortex is believed to contribute to the emergence of adolescent changes in cognition and behavior. While puberty coincides with adolescence, relatively little is known about which aspects of frontal cortex maturation are driven by pubertal development and gonadal hormones. In this review, we highlight existing work that suggests puberty plays a role in the maturation of specific cell types in the medial prefrontal cortex (mPFC) of rodents, and highlight possible routes by which gonadal hormones influence frontal cortical circuit development.

## 1. Introduction

In animals and humans, the behavioral changes that occur during adolescence are accompanied by striking reorganization of neural circuits [1], particularly within frontal cortical regions. Whereas adolescence is a broadly-defined period of cognitive and behavioral transition, puberty is a physiological process that culminates in the attainment of reproductive competence [2]. Though distinct processes, puberty and adolescence are intimately linked through the direct and indirect interactions between gonadal hormones and the brain [3]. In the past decade, there has been a growing interest in the relationship between puberty and the brain and behavioral changes that accompany human adolescence [3,4]. While some aspects of adolescence and frontal cortical function may be unique to humans, a focus on puberty creates opportunities to align and translate mechanistic findings from animals to humans [5]. Animal studies have revealed that puberty influences a broad array of neural processes and circuits that can ultimately shape adult behavior [3,6]. This has largely been assumed to occur via effects of gonadal hormones on subcortical brain function. Here, we review recent evidence from rodent studies that explain how pubertal processes can also sculpt the maturation of the neocortex, including the medial prefrontal cortex (mPFC). These data may help inform understanding of human cognitive development and psychiatric conditions that emerge during adolescence.

**Fig. 1.**
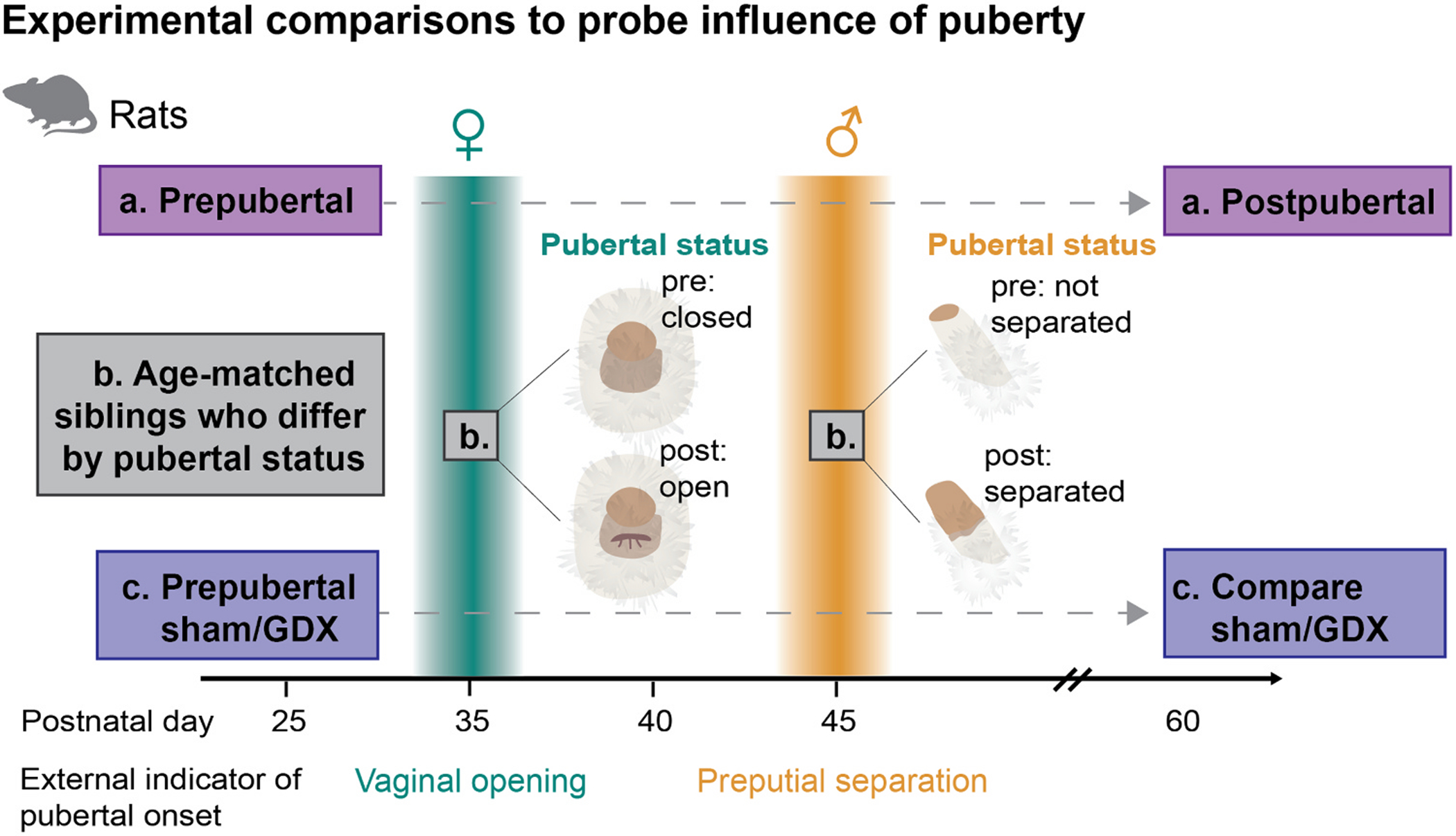
Overview of approaches used to examine the effect of puberty on brain maturation: a) compare prepubertal vs. postpubertal age mice; b) compare siblings who are the same age but differ in their pubertal status; c) compare siblings in adulthood who received sham or gonadectomy surgery prior to puberty onset.

Puberty involves the reactivation of the hypothalamic-pituitary-gonadal (HPG) axis which stimulates the secretion of gonadal hormones from the ovaries and testes. The ovaries primarily secrete estradiol and progesterone whereas the testes primarily secrete testosterone. These gonadal hormones cross the blood brain barrier where they can act via genomic and non-genomic mechanisms to alter brain function. In rats, brain androgen and estrogen receptor expression is comparable between males and females [3,7], and the onset of puberty typically occurs 7–10 days earlier in females than males (Fig. 1). Vaginal opening is the earliest external indicator of puberty in females, triggered by a rise in estradiol. In males, the rise in testosterone secretion is associated with preputial separation. These external indicators allow for the comparison of animals matched in age but discordant for pubertal status, and thus provide an opportunity to disentangle the effects of age and pubertal status on neurodevelopment (Fig. 1c). In this review, we will describe major neurobiological measures in the mPFC that dynamically change during adolescence and examine the potential mechanisms and evidence that pubertal hormones play an influential role. We will reference work that examines the relationship between puberty and brain maturation utilizing three main approaches: a) comparison of pre- and postpubertal age groups, b) comparison of age-matched but pubertal status-discordant siblings, and c) gonad removal prior to puberty onset (with or without hormone replacement). In the existing literature, these three experimental designs have all been used to address the influence of puberty and gonadal hormone exposure on mPFC brain maturation (Fig. 1).

Importantly, gonadal hormones can influence brain structure/function and behavior via two different modes: 1) activational effects that are transient and depend on the hormone being “onboard” at the time of behavioral/neural measurement and 2) organizational effects by which the presence of hormone during a particular developmental window can have long-lasting effects and determine how the brain responds to external inputs or hormones in the future. Adolescence is increasingly recognized as a window of development during which gonadal hormones can establish long-lasting, organizational effects on the brain [6]. A key question is whether peripubertal timing of gonadal hormone exposure is an important factor in sculpting frontal cortex synaptic reorganization during adolescence. If true, then pubertal timing may be a critical factor in development. For example, there may be potential neurodevelopmental consequences of a global trend towards earlier pubertal timing [8], and this may interact with greater organizational influence of gonadal hormones on brain development at younger ages [6].

## 2. Puberty and gray matter maturation of the frontal cortex

*In vivo* structural imaging studies of the neocortex in humans have found that gray matter volume, area, and thickness in medial and lateral frontal areas peak early in adolescence and then decrease [9–11]. Gray matter reduction during adolescence is believed to be driven in large part by the elimination of dendritic spines and their synapses, also known as synaptic pruning [12]. Dendritic spines are the primary site of excitatory synaptic connections on pyramidal neurons, the principal neurons (PNs) of the neocortex [13]. Changes in capillaries [14] and increasing myelination may also play a role in reduced gray matter thickness measures [15]. It is less well known, but adolescence is also a time of stabilization of synapses, as the daily growth and loss of new synapses on dendrites decreases with development [16–18]. Stabilization of dendritic spines may impact neural plasticity and the capacity for circuit reorganization in ways distinct from spine pruning [19,20].

Across species, the approximate age of puberty onset is often an inflection point for spine and synapse density measures within the frontal cortex. Among primates, differences in the timing of synaptogenesis and synapse elimination, as well as changes in gene expression within the prefrontal cortex appear to relate to the species-typical age at sexual maturity [21–23]. However, it is still debated whether or not gray matter thinning and synaptic pruning during adolescence is driven by puberty.

### 2.1. A role for puberty in spine and synapse pruning and dynamics?

Animal models permit manipulation experiments that address the causal relationship between puberty, gonadal hormones, and brain maturation. An earlier study of dorsolateral prefrontal cortex (DLFPC) development, which included data from a few castrated nonhuman primate subjects, suggested that spine pruning on PNs in the DLPFC was not dependent on intact gonads [24]. However, in rodents, a growing body of research demonstrates that gonadal hormones at puberty do influence synaptic pruning within the mPFC in a cell type- and sex-dependent manner. Note, we refer to mPFC to encompass dorsal anterior cingulate (dACC), prelimbic cortex (PL), and infralimbic cortex (IL). When we refer to dorsomedial PFC (dmPFC), we are referring to a region encompassing PL, dACC, and medial portions of M2. While DLPFC in primates and mPFC in rodents are not homologous structures, they receive projections from the mediodorsal thalamus and exhibit overlapping function.

Positive evidence for a role for gonadal hormone in spine/synapse pruning comes from both rats and mice. Drzewiecki et al. showed that synapse number in mPFC, quantified using synaptophysin, peaks around the age of puberty onset in male and female rats (P40 and P35, respectively) [25]. In females but not males, the number of synapses was found to decrease significantly between P35 and P45, suggesting synapse reductions in females were associated with the timing of puberty onset. When the authors compared age-matched siblings who differed in their pubertal status, they found that age-matched (P35) pre- and postpubertal females did not differ in synapse number while postpubertal males at P45 had significantly fewer synapses compared to their age-matched prepubertal siblings [25]. These data suggest that mPFC synapse density in both male and female rats is influenced by pubertal status.

Data published in mice support these correlational findings in rats and add evidence that experimental manipulation of the gonads can block or alter spine pruning in adolescence. However, data from mice suggest this is not the case for all cortical PN cell types. There is likely mixed evidence in the literature because puberty affects mPFC maturation in a cell type- and sex-specific manner [26]. Cortical cell types that differ in their projection targets also differ in gene expression [27–29] and may differ in their maturation [30,31]. Early taxonomies of PNs have focused on two major excitatory pyramidal neuron classes that include intratelencephalic (IT)-type neurons that project to both ipsilateral and contralateral cortex and striatum, as well as ipsilateral amygdala, and pyramidal tract (PT)-type neurons that project subcortically to regions such as the thalamus, midbrain (including the ventral tegmental area), pons, and spinal cord [27,32]. PT-type neurons send collaterals to the ipsilateral striatum, but unlike IT-type neurons, do not target the contralateral hemisphere of cortex or striatum. In addition, IT- and PT-type neurons exhibit distinct morphological and electrophysiological properties [28,33].

Our group recently chose to study mPFC spine pruning on a subpopulation of IT-type neurons that project to the striatum via the corpus callosum, referred to as cross-corticostriatal (cSTR) neurons, because they are specifically implicated in goal-directed learning [34], which has been shown to mature across adolescence [35]. We found that spine pruning on IT-type cSTR neurons did not occur in the absence of intact gonads at puberty in males, suggesting the maturation of this cell type is significantly regulated by puberty in males [36]. In females, prepubertal gonadectomy did not significantly affect average spine density in adulthood, but did significantly increase variance in spine density suggesting possible complex effects [36]. Females also showed higher spine density on this cSTR IT cell type than males at both P29 and P60, suggesting regulation by genetic sex or perinatal organizational effects.

Our group also performed *in vivo* imaging studies of spine pruning and stabilization in mPFC on PT-type neurons, which are brightly labeled with fluorescence in Thy1-YFP transgenic mice [16,37]. Studies of this cell type show significant spine pruning and stabilization occurs over the adolescent transition [16,37]. In females, manipulating ovarian hormones at puberty through prepubertal ovariectomy or prepubertal hormone administration had no significant effect on spine pruning and stabilization (the latter measured by new spine formation and loss) [37]. However, prepubertal ovariectomy and prepubertal hormone administration did significantly affect spine morphological maturation and motility on this PT-type neuron [37].

Layer 2/3 (L2/3) PNs in the cortex are considered IT-type but differ from L5 IT-type neurons by their inputs and outputs, spine density, and morphology. The role of puberty in L2/3 PN spine pruning in mPFC has not been studied in adolescent mice or rats. Studies in adults, however, have shown that adult ovariectomy decreased L2/3 PN spine density in mPFC [38] while estrogen administration increased L2/3 PN spine density in intact and ovariectomized female rats [39,40]. These data indicate that estradiol can affect spine density on L2/3 IT cell types at adult ages but more needs to be done to determine what happens during puberty in this major class of cortical neuron.

#### 2.1.1. A role for puberty in cortical neuron loss in adolescence

The loss of entire neurons, also referred to as neuronal pruning, also contributes to gray matter thinning in the frontal cortex during adolescence [41,42] and strong evidence from a series of experiments suggests that ovarian hormones at puberty play a key role in this process. In rats, researchers found that females, but not males, exhibited significant age-related decreases in neuron number within the mPFC and that this decrease was most pronounced between P35 and P45, the window during which puberty onset occurs [43]. Furthermore, when comparing age-matched (P35) female rats who differed in pubertal status, prepubertal rats exhibited greater neuron numbers in the mPFC compared to their postpubertal siblings [43], suggesting that pubertal hormones may drive neuron loss in development. In a follow up study, neurons were quantified in the mPFC following gonad manipulation. Prepubertal gonadectomy in females but not males was associated with higher neuronal counts compared to their sham surgery siblings, suggesting neuron loss was blocked by gonadectomy in females [44]. Together, these data support a sex-specific role for puberty in driving adolescent period neuron loss in mPFC.

## 3. Microglia and puberty

Microglia are resident immune cells of the central nervous system that have been shown to participate in synaptic pruning [45–47], although the precise mechanisms by which they do so remains an active area of research [48]. A growing body of literature suggests that the function of microglia within hypothalamic regions is sexually differentiated and influenced by gonadal hormone exposure during the perinatal critical period [49]. In adult mice, microglia express low levels of estrogen receptor alpha (ERα) [50] and estradiol has been shown to exert anti-neuroinflammatory effects [51], suggesting that estradiol influences microglial function in the adult brain. However, it is unknown whether the rise in gonadal hormones at puberty contributes to adolescent synaptic reorganization by modulating microglial function. In rats, microglial engulfment of spines on L5 PNs in prelimbic cortex was shown to increase at P39 [52], roughly coinciding with pubertal onset. While male and female rats were included in this study, the authors stated that it was underpowered to detect sex differences and pubertal status was not assessed. It will be informative to perform sex comparison in the future, taking into account the different timing of pubertal onset in males and females.

Understanding the influence of puberty on microglial-mediated synaptic pruning in the frontal cortex could provide insight into adolescent-onset diseases such as schizophrenia in which excessive synaptic pruning has been implicated [53]. Indeed, overexpression of C4 complement protein, a top hit for schizophrenia in a large genome-wide association study [54], leads to increased microglial engulfment of synaptic material within the mPFC during adolescence and reduced synapse density in adult mice [55,56]. At the moment, there is a paucity of data investigating the link between puberty and microglial-mediated synaptic or neuronal pruning within the frontal cortex, although a recent study found evidence for sex-specific microglial pruning of D1 receptors in the nucleus accumbens (NAc) during adolescence [57]. The data outlined above suggest investigating sex-specific mechanisms of microglial pruning during adolescence will be a promising avenue for future research.

## 4. White matter maturation and puberty: focus on the corpus callosum

Human studies indicate that adolescence is a period of white matter expansion driven by a combination of increasing myelination and axonal diameter and the maturation of white matter microstructural properties including density, integrity, and organization. Furthermore, white matter maturation has been associated with pubertal development, in some instances in a sex-dependent manner [58]. The relationship between adrenal and gonadal hormones and white matter maturation in humans has been reviewed elsewhere [4,59]. Here we will focus on insights from rodents about the maturation of the corpus callosum, the major white matter tract that connects the cerebral hemispheres.

In rats, the corpus callosum is larger in males than in females [60], and its myelination continues into adulthood [61]. Prepubertal but not adult ovariectomy has been shown to increase the overall size of the corpus callosum in female rats [62]. In the splenium of the corpus callosum, which carries axons from the visual cortex, prepubertal ovariectomy was associated with a greater number of myelinated axons but no alteration in total axon number [63]. Together, these findings suggest that ovarian hormones coordinate the female-typical pattern of corpus callosum maturation at puberty by reducing axonal myelination. Other data suggest that androgen signaling in early postnatal life contributes to greater myelination and oligodendrocyte density within the corpus callosum that is observed in males [64,65]. One study in male rats found that the number of myelinated axons in the forceps minor of the corpus callosum projecting to L5 of the cingulate cortex increased between juvenile and mid-adolescent time points [66], complementing previously reported increases in white matter volume [43]. Increased myelination was accompanied by a significant increase in conduction velocity measured in acute brain slices [66]. In the future, this approach would be informative when applied to the questions of how age, sex, and pubertal status influence white matter maturation in the corpus callosum.

## 5. Inhibitory neurotransmission in the mPFC

Inhibitory neurotransmission within mPFC is also remodeled during adolescence in a cell type-specific manner [67], and aspects of this process have been found to be sensitive to pubertal manipulation [68]. Several labs have found that inhibitory synaptic transmission onto select populations of mPFC PNs is significantly upregulated during the postpubertal period in mice [31,69] and rats [70] but have not directly tested the role of puberty and or gonadal hormones. However, one recent study demonstrated that the maturation of inhibitory neurotransmission onto L2/3 PNs in dmPFC was accelerated by prepubertal ovarian hormone administration and blocked by ovariectomy in female mice [68]. These data suggest that the maturation of inhibition onto IT-type L2/3 PNs in dmPFC is regulated by pubertal processes.

Several lines of evidence suggest that among the multiple subtypes of cortical inhibitory interneurons (INs), fast-spiking parvalbumin positive (PV+) INs may be the key player in the adolescent maturation of the mPFC. By providing powerful somatic inhibition onto neighboring excitatory PNs [71,72], PV+ INs are positioned to be critical regulators excitation/inhibition balance in mPFC [73]. Within sensory cortices, PV+ IN maturation regulates the closure of sensitive period plasticity [74,75]. Synaptic inputs onto mPFC PV+ INs appear to be remodeled during adolescence within mPFC [76,77], although some of these changes may occur prior to puberty onset [78]. In addition, the intrinsic properties of mPFC PV+ INs exhibit protracted development that extends into adolescence [79,80]. Finally, it is notable that PV+ INs have been implicated in a variety of behaviors that mature over adolescence including cognitive flexibility [80,81], fear learning [82,83], and social behaviors [84–86], although the relationship between PV+ IN maturation and mPFC-dependent behaviors is not necessarily linear.

PV+ INs are a likely site for direct hormonal action on mPFC circuits at puberty because they are the primary neuronal type that expresses estrogen receptor beta (ERß) within the cortex of both males and females [7,87,88]. In males, circulating androgens also have the potential to activate ERß via aromatization of testosterone to estradiol or the metabolism of dihydrotestosterone to 3-beta-diol, an estrogen receptor agonist [89]. While ERß is classically characterized as a nuclear receptor that mediates transcriptional effects, ERß and the closely related ERα also localize to extra-nuclear sites (e.g. axons, dendrites) and have been shown to exert rapid, non-classical effects on the order of minutes to hours [90]. Recent data in adult female rats demonstrate that PV+ neurons in somatosensory cortex are directly modulated by ovarian hormones. Specifically, estradiol increased the intrinsic excitability of L5 PV+ INs through an ERß-dependent mechanism [88]. Whether estradiol exerts similar effects on mPFC PV+ INs in both females and males remains to be tested. Together these data support a working model [91–93] by which gonadal hormones regulate the output of PV+ INs which, in turn, may regulate plasticity and functional development of the mPFC at puberty.

### 5.1. Pubertal regulation of PV expression and presynaptic function

Parvalbumin (PV) expression itself is developmentally regulated, appearing in associative cortices like mPFC (P11–13) later than sensory cortices (P8-11) [94]. During adolescence, PV expression levels within mPFC increase in parallel with the facilitation of excitatory glutamatergic inputs onto these cells [95]. Importantly, PV regulates presynaptic release properties, consistent with its role as a slow calcium buffer. Knockdown of PV expression within mPFC during the periadolescent period significantly reduced spontaneous inhibitory postsynaptic current (sIPSC) frequency onto neighboring L5 PNs, likely through a decrease in presynaptic release probability [97]. Spontaneous excitatory postsynaptic current (sEPSC) frequency onto PV+ INs was also decreased when PV expression was downregulated [97]. These findings point to the homeostatic balance between glutamatergic inputs onto mPFC PV+ INs and their inhibitory output. Whether peripuberty is a sensitive period for setting establishing an excitation/inhibition setpoint is an open question.

#### 5.1.1. Puberty and Perineuronal Nets (PNNs)

**Fig. 2.**
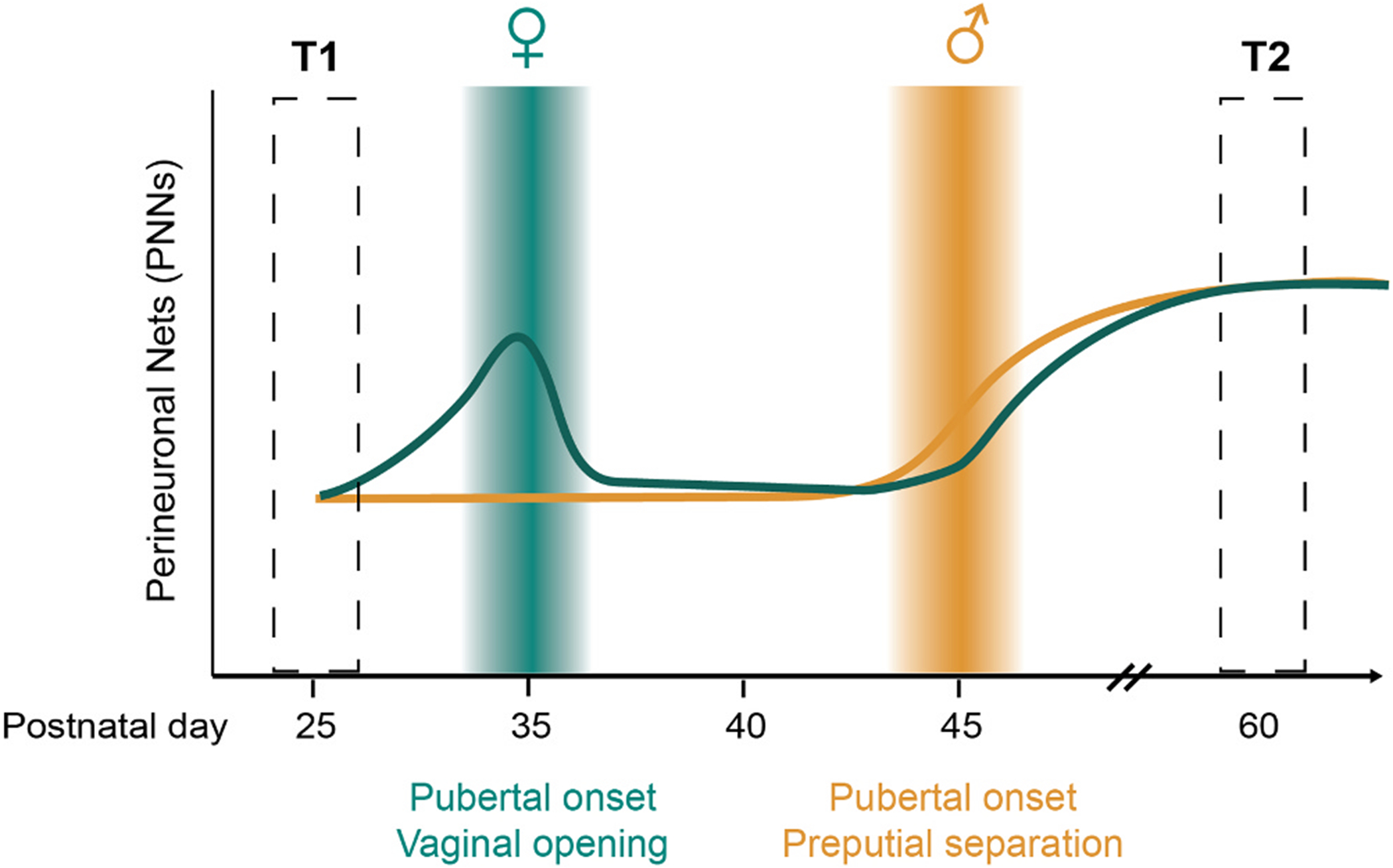
Sex differences in pubertal timing should be considered when determining measurement time points. T1 and T2 indicate juvenile and adult time points that if selected for male and female groups would have missed sex differences in the relationship between puberty onset and PNN number in mPFC observed by Drzewiecki et al., 2020.

Perineuronal nets (PNNs) are extracellular matrix proteins that are important for the maturation of PV+ INs. In addition to their role in synaptic stabilization, PNNs act as cation buffers that can enhance the excitability of PV+ INs [98]. In sensory cortex, PNNs have been shown to regulate the closure of critical periods of plasticity and stabilization of PV networks [99] as well as dynamically regulate learning and plasticity [100]. It remains unclear whether PNNs similarly regulate sensitive period plasticity within the frontal cortex [91]. In mPFC, both male and female rats exhibit a significant increase in the number of PNNs from juvenile to adulthood [101,102]. However, when Drzewiecki and colleagues compared age-matched siblings who differed in their pubertal status, only females showed a significant difference: postpubertal females exhibited an abrupt decrease in the number of PNNs compared to their prepubertal siblings which rebounded after approximately one week [101]. Of note, the sex differences in the temporal dynamics of PNNs surrounding puberty onset would have been missed if males and females were examined at the same pre- and postpubertal ages (e.g. P25 vs. P60) (Fig. 2).

## 6. Maturation of the dopamine system

The dopamine system is unique among neuromodulatory projections to mPFC in that it continues to develop through adolescence. Moreover, while other monoaminergic projections such as serotonin and norepinephrine more broadly innervate rodent neocortex, dopaminergic axons preferentially target the mPFC [103]. This late maturation of dopamine projections to mPFC suggests that dopamine (DA) may have a key role in shaping the structural and functional changes of mPFC associated with adolescence. While the mesocortical DA system is dynamic during the peripubertal period, there is little data examining the influence of gonadal hormone exposure at puberty on the maturation and function of this system. However, we review evidence for adolescent maturation of cortical DA projections as well as data from adult animals showing the influence of gonadal hormones on DA neuromodulation.

### 6.1. Dopaminergic innervation and receptor expression over adolescence

Across mammalian species, the presence of DA axons in the frontal cortex increases during adolescence, innervating all cortical layers and apposing PNs and GABAergic INs [104–106]. In rodents, axons from the ventral tegmental area (VTA) gradually increase innervation of dmPFC until about P60 (early adulthood) in both males and females, with densest innervation of deep cortical layers (L5–6) of anterior cingulate and prelimbic areas [35,107–111]. DAergic axons from VTA appear to be guided through NAc on their way to mPFC by differential expression of DCC, a receptor for the axon guidance cue Netrin-1 [110,112,113]. Finally, evidence suggests that mesocortical DA axons may exhibit greater capacity for activity-dependent potentiation during the adolescent period [114].

Alongside DA axon innervation, dopamine D1 and D2 receptor expression in dmPFC increases rapidly in early adolescence when it peaks and then decreases to adult levels [35,115,116] but see [117]. At the same time, the density of DA appositions on both PNs and GABAergic INs increases, particularly in L5–6 [118,119]. Interestingly, DA receptor subtype expression appears to segregate between IT-type and PT-type PNs: within mPFC L5, D1R-expressing neurons are generally IT-type PNs, while D2R-expressing neurons are PT-type PNs [120,121]. However, only a subset of each of these neuronal classes expresses DA receptors, and expression can vary by cortical layer and projection target [120]. An earlier study in rats reported that D2R mRNA expression localized to corticostriatal and callosal cortico-cortical neurons but not corticopontine, corticothalamic, or corticospinal neurons [122]. This finding is inconsistent with more recent electrophysiological data collected in DA receptor BAC transgenic lines [121] and highlights the importance of using multiple approaches to verify cell type-specific receptor expression and function. Like PNs, INs express both D1R and D2R, with D1R more prevalently expressed [123,124]. Notably, D2R signaling has been shown to differentially modulate PV IN firing in post-adolescent vs. pre-adolescent rats [125]. Further investigation is required to map out the developmental trajectory of DA receptor expression in specific subsets of PNs and INs which may produce complex functional effects on mPFC activity across development [126].

#### 6.1.1. Influence of gonadal hormones on dmPFC dopamine

Immunohistochemical studies examining expression of tyrosine hydroxylase (TH), the enzyme responsible for generating the common precursor for dopamine and norephinephrine, have shown that TH+ fiber density increases in mPFC over adolescence, while the density of fibers expressing dopamine beta-hydroxylase (DBH), which converts dopamine to norepinephrine, remains stable [35]. Perinatally gonadectomized male rats exhibit reduced TH+ fibers compared to sham-operated rats [127], suggesting a role for sex hormones in establishing DA innervation. Studies in adult animals further suggest gonadal hormones regulate mPFC DA. In female rats, basal mPFC DA concentration varies across the estrous cycle, with lowest DA concentration during proestrus, the phase at which estradiol levels peak [128]. However, ovariectomized rats treated with estradiol [129] or the ERß agonist DPN [130] exhibit increased concentrations of mPFC DA compared to vehicle controls. The influence of gonadal hormones on basal mPFC DA in adult males appears complex: shortly after gonadectomy (4 days) males display a reduction in TH+ fibers in dACC [131] and lower basal DA levels that are recovered by supplemental testosterone or estradiol administration [132]. However, at longer post-surgical durations (28 days) gonadectomy was associated with higher than normal levels of TH+ fiber innervation [131] and elevated basal DA levels in dACC that were both normalized by testosterone but not estradiol administration [132]. An important regulator of mPFC DA reuptake and metabolism is catechol-O-methyltransferase (COMT) [133]. Ovariectomy increased mPFC COMT expression relative to intact females, and estradiol administration to intact male rats reduced COMT expression in mPFC relative to vehicle-treated males [134]. Together, these data suggest that in adulthood estradiol increases DA release and reuptake in mPFC of both sexes, whereas testosterone may play a complex role in maintaining mPFC DAergic tone in males.

Literature examining the influence of sex hormone exposure on mPFC DA function during puberty is rare. One notable study showed that rats of the same age but different pubertal status have no difference in TH+ fibers in the mPFC, indicating that developmental changes in dopamine axon innervation may not be dependent on puberty [111]. However, this study did not examine what fraction of TH+ fibers were also DBH+ (confounding DA related signal with NE related signals). More work is needed to understand how pubertal hormones may influence the maturation of DA neuromodulation in mPFC during adolescence and will be aided by genetic and viral tools to selectively label and manipulate DAergic projections.

## 7. Interacting systems and a working model

**Fig. 3.**
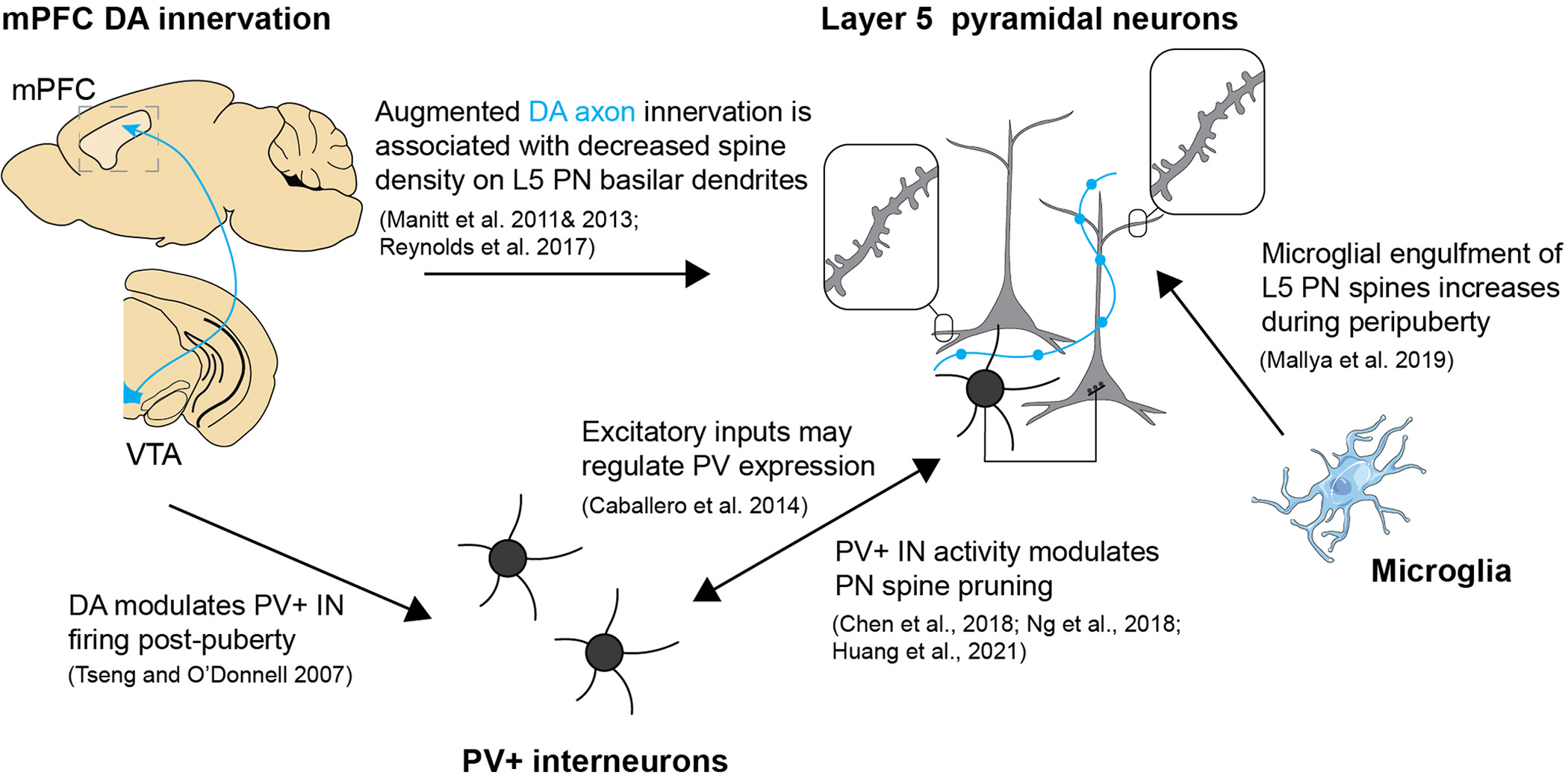
Interacting systems within mPFC during pubertal development.

Because gonadal hormones have such wide reach throughout the body and nervous system, it is difficult to isolate the influence of puberty on a given cellular or synaptic process. Furthermore, the dynamic neurobiological systems we have described during adolescence are known to significantly influence one another. Such interactions may confound our attempt to identify which processes are puberty dependent versus independent. These are difficult challenges to overcome, but we outline routes by which gonadal hormones may impact mPFC maturation (Table 1 and Fig. 3) through direct effects on microglia, PV+ INs, and DA neurons, which may act alone or synergistically to regulate excitability, synaptic strength and synaptic spine density. It is important to note that these effects are also not likely to be global but circuit and cell type-specific. We suggest, based on current evidence, that within mPFC, IT-type PNs may be the most influenced by gonadal hormone levels at puberty, while PT-type PNs may be more independent. Further data on cell subtypes may reveal even greater specificity.

## 8. Conclusions and future directions

Growing evidence from rodent studies suggests that the rise in gonadal hormones at puberty plays a significant role in sculpting the development of the mPFC. Spine and synapse pruning is a hallmark of frontal cortex maturation and, in rodents, there is evidence that this pruning is initiated at puberty [25]. Furthermore, in male mice, adolescent spine pruning on a specific class of PN is dependent on intact gonads at puberty [36]. In female mice there is evidence that gonadal hormones alter the maturation of inhibitory synapses in the dmPFC [68], and recent data suggests that changes in inhibitory transmission and synaptic pruning could occur downstream of estrogenic modulation of PV+ INs [88]. In addition to PV+ INs, gonadal hormones may influence mPFC development via actions on microglia and the dopamine system. While much more work is needed, current data suggest that cell type- and circuit-driven approaches to studying gonadal hormone action in the frontal cortices will be highly informative. We anticipate translation from rodents to humans will be significantly advanced if we can map specific cell types to circuits that can be isolated using non-invasive human neuroimaging techniques.

While the role of puberty and pubertal timing in human development remains poorly understood, these topics are of high translational relevance. In humans, puberty onset is occurring at earlier ages, and precocious puberty in girls is associated with worse mental health outcomes, particularly for anxiety and depression [138]. Furthermore, access to gender affirming hormone therapies is increasingly common, including among peripubertal youth [139]. Finally, puberty onset is a time when sex biases in psychiatric risk emerge. Thus, we need more specific information about when, where, and how gonadal hormones influence the frontal cortex to better understand these important phenomena and promote the healthy transition to adulthood.

## Figures and Tables

**Table 1:** Interacting systems within mPFC during pubertal development.

Interaction	Effect	Citations	Comments/Caveats
Gonadal hormones influence microglia	Microglial engulfment of spines on L5 PNs in prelimbic cortex increases at peripuberty	[52]	
Microglia influence dendritic spine density	Increased recruitment of C4-dependent microglial-mediated pruning during adolescence reduces adult spine density	[55,56]	
Gonadal hormones influence PV+ INs	Estradiol increases intrinsic excitability of L5 PV+ INs in an ERß-dependent manner	[88]	Not demonstrated in mPFC, but ovarian hormones increase inhibitory synaptic transmission onto L2/3 PNs in mPFC [68]
PV+ INs influence dendritic spines	Increasing PV+ interneuron activity in frontal cortex reduces stress-induced loss of dendritic spines	[135–137]	
Gonadal hormones influence DA	Adult castration augments dmPFC DAergic fiber innervation and basal DA levels in males. Adult ovariectomy reduces basal mPFC DA and is counteracted by estradiol administration.	[129,131,132]	Data lacking for mPFC during peripubertal period
DA influences dendritic spines	Ectopic overexpression of DAergic fibers in adult mPFC is associated with reduced spine density on basilar dendrites of L5 PNs	[110,112]	
DA influence PV+ INs	D2R enhancement of PV+ interneuron excitability emerges postpuberty	[125]	Subject sex was not indicated in this study, and causal role of puberty was not demonstrated
